# Supplementary material for: Novel Driver Strength Index highlights important cancer genes in TCGA PanCanAtlas patients
Source: PeerJ. 2022 Aug 11;10:e13860. doi: 10.7717/peerj.13860 (PMC9375969; doi:10.7717/peerj.13860)
Supplement: Supplemental Information 2 [file peerj-10-13860-s002.zip › COHORTS/genes plots/cohorts/2021_8_16_14_9_distribution_cohorts_MESO.pdf]

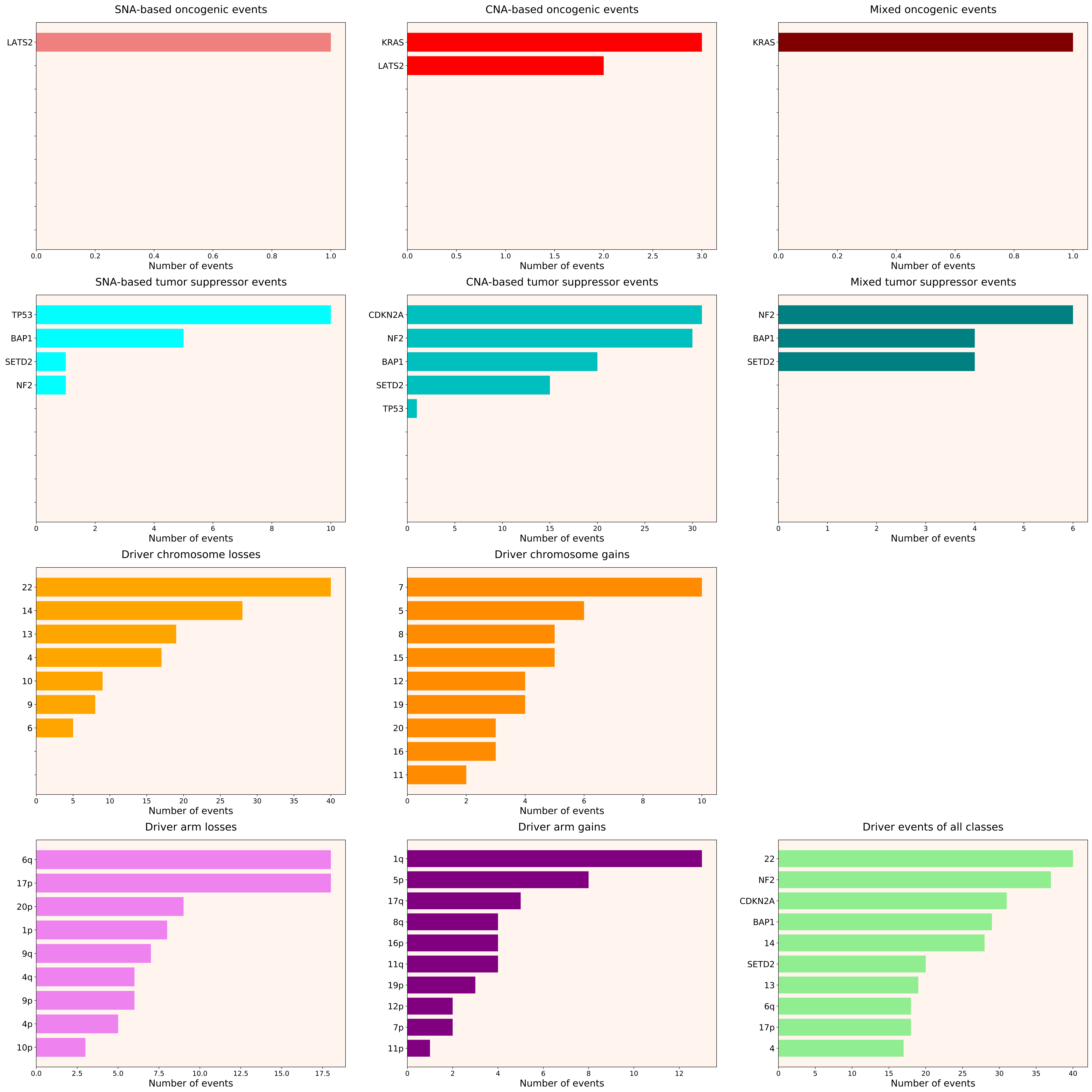
SNA-based oncogenic events
LATS2
0.0 0.2 0.4 0.6 0.8 1.0
Number of events
CNA-based oncogenic events
KRAS
LATS2
0.0 0.5 1.0 1.5 2.0 2.5 3.0
Number of events
Mixed oncogenic events
KRAS
0.0 0.2 0.4 0.6 0.8 1.0
Number of events
SNA-based tumor suppressor events
TP53
BAP1
SETD2
NF2
0 2 4 6 8 10
Number of events
CNA-based tumor suppressor events
CDKN2A
NF2
BAP1
SETD2
TP53
0 5 10 15 20 25 30
Number of events
Mixed tumor suppressor events
NF2
BAP1
SETD2
0 1 2 3 4 5 6
Number of events
Driver chromosome losses
22
14
13
4
10
9
6
0 5 10 15 20 25 30 35 40
Number of events
Driver chromosome gains
7
5
8
15
12
19
20
16
11
0 2 4 6 8 10
Number of events
Driver arm losses
6q
17p
20p
1p
9q
4q
9p
4p
10p
0.0 2.5 5.0 7.5 10.0 12.5 15.0 17.5
Number of events
Driver arm gains
1q
5p
17q
8q
16p
11q
19p
12p
7p
11p
0 2 4 6 8 10 12
Number of events
Driver events of all classes
22
NF2
CDKN2A
BAP1
14
SETD2
13
6q
17p
4
0 5 10 15 20 25 30 35 40
Number of events
